# Salinity and ABA Seed Responses in Pepper: Expression and Interaction of ABA Core Signaling Components

**DOI:** 10.3389/fpls.2019.00304

**Published:** 2019-03-19

**Authors:** Alessandra Ruggiero, Simone Landi, Paola Punzo, Marco Possenti, Michael J. Van Oosten, Antonello Costa, Giorgio Morelli, Albino Maggio, Stefania Grillo, Giorgia Batelli

**Affiliations:** ^1^National Research Council of Italy, Institute of Biosciences and Bioresources (CNR-IBBR), Reaserch Division Portici, Portici, Italy; ^2^Department of Agriculture, University of Naples “Federico II”, Portici, Italy; ^3^Council for Agricultural Research and Economics, Research Centre for Genomics and Bioinformatics (CREA-GB), Rome, Italy

**Keywords:** *Capsicum annuum* L., abscisic acid, *PYR/PYL/RCARs*, *PP2Cs*, *SnRK2s*, seed germination, seed viability

## Abstract

Abscisic acid (ABA) plays an important role in various aspects of plant growth and development, including adaptation to stresses, fruit development and ripening. In seeds, ABA participates through its core signaling components in dormancy instauration, longevity determination, and inhibition of germination in unfavorable environmental conditions such as high soil salinity. Here, we show that seed germination in pepper was delayed but only marginally reduced by ABA or NaCl with respect to control treatments. Through a similarity search, pepper orthologs of ABA core signaling components *PYL* (*PYRABACTIN RESISTANCE1-LIKE*), *PP2C* (*PROTEIN PHOSPHATASE2C*), and *SnRK2* (*SUCROSE NONFERMENTING1 (SNF1)-RELATED PROTEIN KINASE2*) genes were identified. Gene expression analyses of selected members showed a low abundance of *PYL* and *SnRK2* transcripts in dry seeds compared to other tissues, and an up-regulation at high concentrations of ABA and/or NaCl for both positive and negative regulators of ABA signaling. As expected, in hydroponically-grown seedlings exposed to NaCl, only PP2C encoding genes were up-regulated. Yeast two hybrid assays performed among putative pepper core components and with *Arabidopsis thaliana* orthologs confirmed the ability of the identified proteins to function in ABA signaling cascade, with the exception of a CaABI isoform cloned from seeds. BiFC assay *in planta* confirmed some of the interactions obtained in yeast. Altogether, our results indicate that a low expression of perception and signaling components in pepper seeds might contribute to explain the observed high percentages of seed germination in the presence of ABA. These results might have direct implications on the improvement of seed longevity and vigor, a bottleneck in pepper breeding.

## Introduction

Pepper (*Capsicum* spp.) is an economically important genus of the Solanaceae family, with a global production of 38.4 million tons including green fruits and dried pods, harvested from 3.7 million hectares in 2016 ^1^. In sweet pepper (*Capsicum annuum* L.), seed vivipary and short longevity are two major challenges of commercial production (Marrush et al., 1998; Lanteri et al., 2000; De, 2004; Sano et al., 2016).

Abscisic acid (ABA) plays a major role in the adaptation to stresses during the vegetative phase, as well as in the establishment of seed dormancy, seed longevity, and inhibition of germination under unfavorable conditions (Zhu, 2002; Finkelstein, 2013; Ruggiero et al., 2017). Seed germination starts with the uptake of water by imbibition of the dry seed, followed by embryo expansion. The uptake of water is triphasic with a rapid initial uptake (phase I, i.e., imbibition) followed by a plateau phase (phase II). A further increase in water uptake (phase III) happens only when germination occurs, as the embryo axis elongates and breaks through the covering layers, typically the endosperm and the testa (Manz et al., 2005). While in *Arabidopsis thaliana* testa and endosperm rupture occur simultaneously, in many *Solanaceae* seeds these two events are temporally distinct (Petruzzelli et al., 2003). Mechanical resistance from testa and endosperm dormancy appears to be the cause of nondeep physiological dormancy in seed model systems such as *A. thaliana* and *Solanaceae* species (Leubner-Metzger, 2003). Enzymes that facilitate testa rupture can be released by the endosperm and/or the radicle. β-1,3-glucanases (βGlu) facilitate endosperm rupture by breaking intercellular adhesion and causing cell separation. In pepper, the accumulation of βGlu occurs prior to radicle emergence. Unfavorable osmotic potentials, darkness and ABA inhibit endosperm rupture and βGlu accumulation in the micropylar cap of the seeds, the site of radicle emergence (Petruzzelli et al., 2003).

In non-dormant seeds, exogenous ABA also inhibits the transition from water uptake phase II to III and late embryo cell expansion, but does not affect phase I and II and testa rupture (Müller et al., 2006).

In *Arabidopsis*, ABA inhibition of germination is mediated by members of the core ABA signaling pathway as well as by ABF-type transcription factors.

Components of this signaling pathway have been isolated in the past decades, and include the PYRABACTIN RESISTANCE (PYR)/PYL (PYR1-LIKE)/REGULATORY COMPONENT OF ABA RESPONSE (RCAR), (hereafter referred to as PYLs), PROTEIN PHOSPHATASE2C (PP2C), and SNF1-RELATED PROTEIN KINASE2 (SnRK2) (Ma et al., 2009; Park et al., 2009). In the absence of ABA, clade A PP2Cs, a family of major negative regulators of ABA responses, bind to and inhibit SnRK2 kinases. The association keeps the kinases inactive by blocking their catalytic cleft and by dephosphorylating the activation loop (Soon et al., 2012). In response to environmental or developmental cues, ABA is perceived by PYL receptors and induces closure of two highly conserved β-loops that function as a gate and latch, and lock the pocket, creating a binding surface for the PP2Cs (Cutler et al., 2010; Moreno-Alvero et al., 2017). A conserved tryptophan residue in the PP2C inserts directly between the gate and latch, and functions to further lock the receptor in a closed conformation (Melcher et al., 2010; Santiago et al., 2012; Moreno-Alvero et al., 2017). SnRK2 kinases are thus released from PP2C inhibition, and can then phosphorylate many downstream effectors (Fujii et al., 2009), including the basic leucine-zipper transcription factors such as ABI5 (ABSCISIC ACID INSENSITIVE 5). A double *snrk2.2/2.3* mutant displayed highly ABA-insensitive germination and increased seed dormancy (Fujii et al., 2007). Seed germination was resistant to 50-100 μM ABA in a sextuple *pyr1/pyl1/2/4/5/8* mutant (Gonzalez-Guzman et al., 2012). Imbibed *abi5* mutant seeds were able to perform the first stages of seed germination, but were arrested before the radicle penetration of the inner testa and endosperm (Lopez-Molina et al., 2002).

Genomic tools are now available to analyze the molecular details of these phenomena in pepper (Kim et al., 2014; Qin et al., 2014; Kim et al., 2017; Hulse-Kemp et al., 2018). The elucidation of the core ABA signaling pathway in model systems and the identification of key secondary regulatory proteins allows this knowledge to be applied to crop species (Klingler et al., 2010; Hou et al., 2016). Previous work has already characterized core components in *Solanum lycopersicum* (Gonzalez-Guzman et al., 2014; Chen et al., 2016) and a pair of pepper PP2C/PYL proteins has been previously functionally analyzed (Lim and Lee, 2016). Different studies have shown the applications of ABA signaling core components to enhance plant stress tolerance, through either genetic engineering or chemical approaches in crops (Okamoto et al., 2013; Rodriguez and Lozano-Juste, 2015; Zhang et al., 2016; Vaidya et al., 2017). Pepper presents a potential practical application of these methods for genetic improvement that can directly benefit breeders, producers, and consumers.

Here, we report on ABA and NaCl sensitivity in pepper seeds and plants as well as the identification of components of the *C. annuum* *PYL*, *PP2C*, and *SnRK2* gene families. We have characterized pepper’s sensitivity to ABA and NaCl at the germination stage by evaluating gene expression and the potential for *in vivo* interaction among the ABA signaling components. We performed yeast two hybrid assays among putative pepper core components and with *Arabidopsis* orthologs to confirm the ability of these proteins to function in the ABA signaling cascade. We also verified interactions in protoplasts using a bi-molecular florescence assay.

## Materials and Methods

### Plant Material

*Capsicum annuum* L. Quadrato D’Asti giallo (2480) seeds were provided by “S.A.I.S. Spa” (Italy), while seeds of the genotypes Corno di Toro rosso (QSB294TS), (refer to as Corno rosso), Corno di Toro giallo (QSB296TS) (refer to as Corno giallo), Friariello (Nocera selection, PGM283), Nocera rosso (Japanese selection, QPA1909TS), Nocera giallo (Japanese selection, QPA1310TS), and Marconi rosso (R15020) were kindly gifted by “La Semiorto Sementi s.r.l.” (Italy).

For germination analyses, seeds were sown on solid MS medium (1X MS Salts including vitamins, 15 g/L Plant agar, pH 5.7) in the presence of ABA (1, 5, and 10 μM) or NaCl (25, 50, and 100 mM). Germination was scored daily in terms of radical emergence and fully expanded cotyledons.

For seedling sensitivity to ABA and NaCl, seedlings were transferred from germination media after 9 days and were grown on vertical plates on solid MS medium (1X MS Salts including vitamins, 5 g/L Sucrose, 15 g/L Plant agar, pH 5.7) in the presence of 100 mM NaCl or 20 μM ABA. Root length was scored every 2 days, shoot weight and photographs were taken after 5 days of growth.

In hydroponic culture, pepper seedlings were grown in solution containing: 1.5 mM Mg(NO_3_)_2_⋅6H_2_O, 3.4 mM Ca(NO_3_)_2_⋅4H_2_O, 1 mM KNO_3_, 1.8 mM K_2_SO_4_, 1.5 mM KH_2_PO_4,_ and 14 mg/L Hidromix (Valagro, Italy), pH 6.2. The solution was changed weekly. After 17 days of culture, NaCl 200 mM was added to the solution to impose salt stress. After 3 h of salt treatment, shoots and roots of each condition were collected separately.

### RNA Isolation, cDNA Synthesis, and qRT-PCR

Total RNA was extracted from seeds, shoots and roots (100 mg) using RNeasy^®^ Plant Mini kit (Qiagen, Germany) following the manufacturer’s instructions. RNA quantity was measured spectrophotometrically by NanoDrop ND-1000 Spectrophotometer (NanoDropTechnologies, United States), and integrity was verified on a denaturing agarose gel. One microgram of total RNA was DNase-treated and reverse transcribed using QuantiTect^®^ Reverse Transcription kit (Qiagen, Germany) according to manufacturer’s instructions. For gene expression analyses, the complementary DNA was diluted 1:20 and 2 μL of diluted cDNA were used for each qRT-PCR reaction, performed with 6.25 μL of 1X Platinum^®^ SYBR^®^ Green qPCR SuperMix (Thermo Fisher Scientific, United States) and 1.75 μL of primer mix (4.28 μM) in a 12.5 μL PCR reaction. Primers used are listed in Supplementary Table S1. Reactions were performed with ABI 7900 HT (Applied Biosystems, United States). Cycling conditions were: 10 min at 95°C, followed by 40 cycles of 95°C for 15 s and 60°C for 1 min. Three or four biological replicates per treatment, each with three technical replicates were tested. PCR product melting curves were analyzed to confirm the presence of a single peak, indicative of one PCR product per primer couple assayed. For relative quantification of gene expression, *Capsicum annuum* eukaryotic initiation factor 5A2 (*EIF5A2*) (Acc.no. AY484392) was used as endogenous reference since its expression was found stable in all the analyzed tissues and treatments as also reported by Wan et al. (2011). Quantification of gene expression was carried out using the 2^-ΔΔCt^ method (Livak and Schmittgen, 2001) and reported as relative expression levels, compared to control conditions as internal calibrator.

For absolute qRT-PCR total RNA was extracted from dry seeds, germinating seeds (i.e., seeds incubated for 4 days on control medium), shoots and roots from seedlings grown in hydroponic system in control condition. RNA isolation, cDNA Synthesis and qRT-PCR were performed as already mentioned above. All primer pairs (reported in Supplementary Table S1) were tested by PCR. A single product of the correct size for each gene was confirmed by agarose gel electrophoresis. The amplified fragment of each gene was subcloned into the pGEM^®^-T Easy vector (Promega, United States) and used to generate standard curves by serial dilutions. Results were analyzed using the ABI PRISM 7900HT Sequence Detection System, Version 2.3. Analysis of variance (ANOVA) on absolute qRT-PCR data was carried out using the SPSS software package (SPSS 19 for Windows, SPSS Inc., an IBM Company, United States). When ANOVA indicated that a single factor or their interaction was significant, mean separation was performed using the Duncan’s multiple range test at *p* < 0.05 on each of the significant variables measured.

### Yeast Two-Hybrid Assay

For yeast two-hybrid experiments, the prey plasmid pGADT7 and the bait plasmid pGBKT7 (Clontech, United States) were used. The full-length coding sequence of *CaABI* and *CaHAI* were PCR amplified using RNA prepared from seeds and cloned in frame into pGADT7 between *Eco*RI and *Xho*I, *SmaI* and *XhoI* restriction sites, respectively. The full-length coding sequence of *CaPYL2* and *CaPYL4* were PCR amplified using RNA prepared from leaves and cloned in frame with the GAL4 binding domain (BD) of pGBKT7 digested with *SmaI* and *PstI*. The full-length coding sequence of *CaPYL8*, *CaSnRK2.3*, and *CaSnRK2.6* were PCR amplified using RNA prepared from seeds and cloned in frame into pGBKT7 between *Eco*RI and *BamH*I, *Eco*RI and *Sal*I, *Eco*RI, and *BamH*I restriction sites, respectively. Plasmids were sequenced to rule out PCR-induced mutations. Primers used for PCR amplification of the mentioned genes are listed in Supplementary Table S2. The bait and prey plasmids containing *Arabidopsis thaliana* genes were previously described (Park et al., 2009; Hou et al., 2016).

The bait and prey plasmids were transformed into yeast strain AH109 (Clontech, United States) using the Lithium acetate/Polyethylene glycol method (Bai and Elledge, 1997). The self-activation test was performed prior to the testing of combinations of interest as reported in Docimo et al. (2016). After verifying that the bait and prey plasmids were not showing self-activation, co-transformations to verify interactions were performed. Transformed colonies containing bait and prey plasmids were selected on synthetic drop-out medium lacking tryptophan and leucine (-W/-L). Co-transformants were grown overnight in liquid culture lacking tryptophan and leucine (-W/-L). For the interaction between bait and prey, an equal amount of cells was spotted on medium lacking tryptophan, leucine, histidine and adenine (-W/-L/-H/-A). Positive and negative controls were also performed as indicated in the figure legend.

### Bimolecular Fluorescence Complementation Assay

The CDS of *CaPYL2* and *CaPYL4* were fused downstream of N-terminal region of YFP, while *CaHAI* was fused downstream of the C-terminal region of YFP, using pUGW0 vectors (Nakagawa et al., 2007). Leaf protoplasts were prepared and transformed according to Pedrazzini et al. (1997), using 3 weeks old *N. tabacum* plants. DNA (40 μg of each construct) was introduced into 1 × 10^6^ protoplasts by PEG-mediated transfection. After 16 h incubation in the dark at 25°C, each interaction was split in two and one was treated with 50 μM of ABA. After the incubation time, YFP fluorescence in protoplast cells was detected by confocal microscopy.

### Confocal Imaging

Confocal microscopy analyses were performed on an Inverted Z.1 microscope (Zeiss, Germany) equipped with a Zeiss LSM 700 spectral confocal laser-scanning unit (Zeiss, Germany). Samples were excited with a 488 nm, 10 mW solid laser with emission split at 505 nm for YFP.

### Bioinformatics

Sequences of pepper (*Capsicum annuum*) ABA receptors (PYL/PYR/RCAR), PP2Cs and SnRK2s were found using a multiple database search to identify potential members of these families. Tomato (*Solanum lycopersicum*) and *Arabidopsis thaliana* sequences were previously identified by other authors (Schweighofer et al., 2004; Gonzalez-Guzman et al., 2014) and obtained at https://solgenomics.net and https://www.arabidopsis.org, respectively. Pepper sequences were obtained using a BLAST P approach at http://pepperhub.hzau.edu.cn/pegnm/ database, using each *A. thaliana* and tomato sequence as queries. The obtained hits were filtered using a blast score cut-off ≥ 150. Alignments and phylogenetic analyses were performed using the software MEGA version 6 (Tamura et al., 2013). Sequence alignment was achieved using the MUSCLE algorithm, using a maximum number of interactions equal to 32. Phylogenetic trees were constructed using the maximum likelihood method with the substitution JTT model gamma distributed. The test of phylogeny was performed using the bootstrap method with a number of replications equal to 100.

For cloned PP2Cs, alignments of multiple amino acid sequences were carried out using Clustal W^2^. The alignment results were marked using BOXSHADE 3.21 software^3^.

## Results

### Pepper Seed Germination in Presence of ABA and NaCl

**FIGURE 1:**
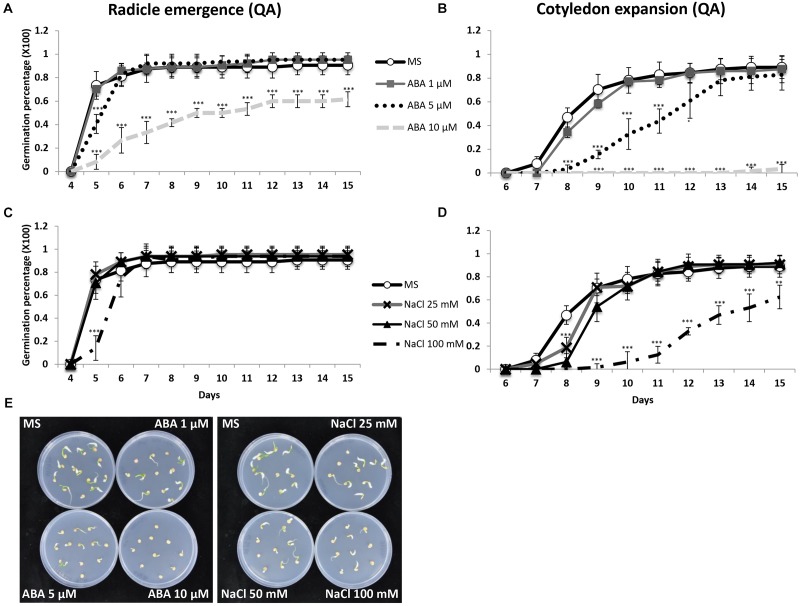
Daily germination percentage in terms of radicle emergence **(A,C)** and cotyledon expansion **(B,D)** of QA seeds treated with different concentrations of ABA or NaCl. **(E)** QA seeds incubated (day 8) on control medium and media with different concentrations of ABA (1, 5, 10 μM) or NaCl (25, 50, 100 mM). Values indicate mean ± SD (*n* = 50). Asterisks represent significance levels using Student *t*-test; ^∗^*P* ≤ 0.05; ^∗∗^*P* ≤ 0.01; ^∗∗∗^*P* ≤ 0.005. QA, Quadrato D’Asti giallo.

To verify the sensitivity of *Capsicum annuum* to abiotic stress and ABA treatments at the seed stage, we scored the germination percentage of commercial seeds of Quadrato D’Asti giallo (QA), a high yielding variety among the most commonly cultivated for marketing in Italy and abroad. The germination rates in terms of radicle emergence and cotyledon expansion of seeds in presence of ABA or NaCl were analyzed in detail (Figure 1). After 15 days, seeds treated with 1 or 5 μM ABA reached virtually 100% germination, while those treated with 10 μM ABA showed 62% seed germination (Figure 1A). The seeds incubated with 10 μM ABA had complete inhibition of cotyledon expansion, while the presence of 5 μM ABA resulted in a 6 day delay compared to controls or 1 μM ABA (Figure 1B). The radicle emergence was not affected by salt treatments except for 100 mM NaCl, which caused a 1 day delay of germination compared to no or lower NaCl concentrations (Figure 1C). Sodium chloride also delayed cotyledon expansion. At 100 mM NaCl, only 63% of the seedlings had fully expanded cotyledons after 15 days of incubation, while at 0, 25, and 50 mM 90% of the cotyledons were fully expanded (Figure 1D). The delayed cotyledon expansion caused by the highest concentrations of ABA and NaCl was also visible early as 8 days (Figure 1E). Therefore, the presence of NaCl or ABA had little effect on QA seed germination at low and medium concentrations with a clear effect only at high concentrations. A similar behavior was observed in other tested varieties, which showed significant germination reduction at 10 μM ABA and 100 mM NaCl (Supplementary Figure S1).

**FIGURE 2:**
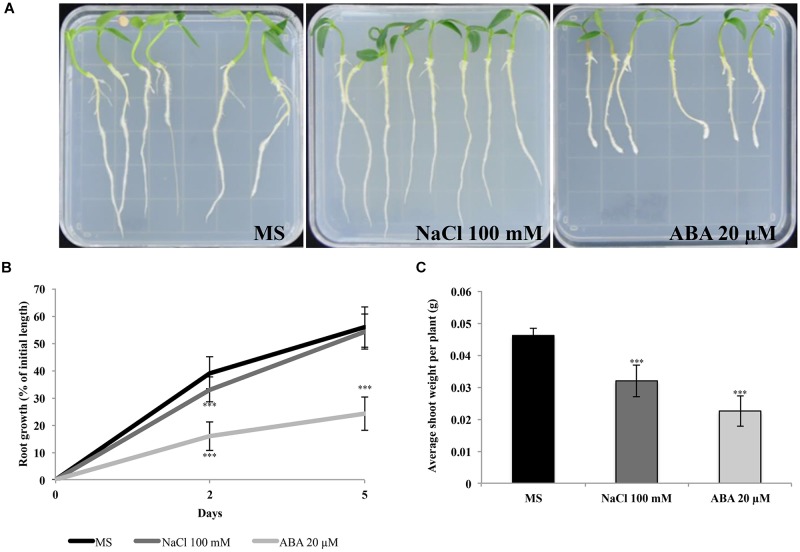
QA seedling sensitivity to ABA and NaCl. **(A)** Representative plates of seedlings grown on control, NaCl and ABA media. **(B)** QA seedling root growth measured as percentage of initial length. **(C)** Shoot weight (normalized per plant). Seedlings were transferred from germination media after 9 days and were grown on vertical plates for 5 days in the presence of 100 mM NaCl or 20 μM ABA. Values indicate mean ± SD (*n* = 24). Asterisks represent significance levels using Student *t*-test; ^∗∗∗^*P* ≤ 0.005. QA, Quadrato D’Asti giallo.

We also tested response of seedlings to ABA and NaCl to verify sensitivity at different developmental stages. We therefore scored root growth of QA seedlings germinated on control media and subsequently transferred to plates containing NaCl or ABA for 5 days (Figure 2). As shown in Figure 2A,B, root growth was inhibited at 20 μM ABA. In particular, 2 days after transfer, the seedlings grown in presence of 20 μM ABA showed a 16% increase of the initial root length compared to a 33% increase in 100 mM NaCl and a 40% increase in plants grown in the control medium. After 5 days of incubation, there was a 30% difference in root growth between seedlings grown on ABA and no or NaCl treatments. Measurements of shoot weight at the end of the experiment allowed for a discrimination of the treatments, showing a reduction in plants treated with NaCl 100 mM or ABA 20 μM compared to controls (Figure 2C).

### *Capsicum annuum* *PYL*, *PP2C*, and *SnRK2* Identification

**FIGURE 3:**
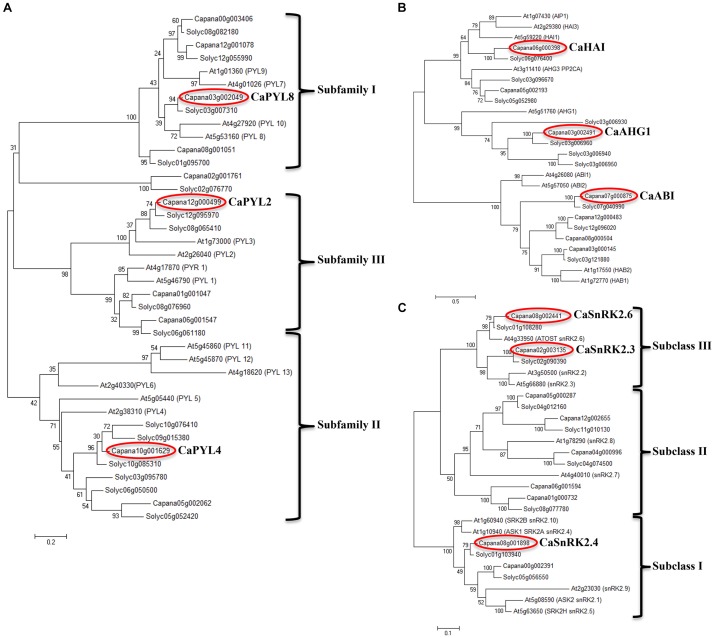
Unrooted phylogenetic trees of the core components in ABA signaling: PYR/PYL/RCAR (*PYL*) **(A)**, clade A *PP2C* **(B)**, and *SnRK2* **(C)** gene families. Trees were drawn using the maximum likelihood method in the MEGA V6 software. Red circles indicate genes selected for further experiments.

Using annotated tomato and *Arabidopsis* protein sequences, we identified putative orthologs in pepper genome (*C. annuum* L_Zunla-1, Qin et al., 2014) of ABA signal transduction core components: PYR/PYL/RCAR (PYLs), clade A PP2Cs and SnRK2s (Figure 3). A high bootstrap was observed for the three analyzed families, with *C. annuum* homologs showing a closer similarity to the tomato counterparts. Ten *PYL* genes (Figure 3A) were identified and clustered in the classical three subfamilies (Ma et al., 2009), with the exception of *Capana02g001761*, which clustered with *Solyc02g076770*. A multiple aminoacid sequence alignment of Ca- and AtPYLs showed that functional residues in the conserved loops (CL1-CL4) were well conserved (Supplementary Figure S2).

Seven genes encoding putative clade A *PP2Cs* showed a partition in 2 subfamilies (Figure 3B); the first included *Capana06g000398*, *Capana05g002193* and *Capana03g002491*, clustering with *Arabidopsis* *AIP1*, *HAI3*, *HAI1*, *AHG3*, and *AHG1*; *Capana03g000145*, previously characterized as *CaADIP1* grouped with *Arabidopsis* *ABI1*, *ABI2*, *HAB2*, and *HAB1*, as previously shown (Lim and Lee, 2016) and with *Capana07g000875*, *Capana12g000483*, and *Capana08g000504*. We focus our studies on three CaPP2Cs representatives: *Capana03g002491*, *Capana06g000398*, and *Capana07g000875* (circled in red, Figure 3B). A multiple alignment of Capana03g002491 deduced amino acid sequences with AtAHG1, Solyc03g006960, AtABI1 and AtHAB1 showed that most of the functional residues were well conserved, including those necessary for the Mn/Mg^++^ interaction and the interaction with PYLs (Supplementary Figure S3). Similarly to AtAHG1, Capana03g002491 lacks the tryptophan that in other clade A PP2Cs participates to the binding with ABA (W385 in AtHAB1, W300 in AtABI1), which in *Arabidopsis* appears to be a unique feature of AHG1. Thus, we named Capana03g002491 as CaAHG1 (Figure 3B).

To study protein interactions, *Capana06g000398* and *Capana07g000875* were isolated and cloned using RNA extracted from seeds. The proteins encoded by the cloned coding sequences showed only a few conservative amino acid replacements with similar properties (Supplementary Figure S4). A notable exception is the substitution of a phenylalanine involved in the Van der Waal contacts with PYLs (F306 in ABI1, Yin et al., 2009), which in sequences from Solanaceous plants is replaced by a serine (S), indicating possible modifications in the binding with regulatory PYLs. Furthermore, the cloned *Capana07g000875*, which corresponds to XM_016724784.1, one of the four predicted splicing variants of this gene, has a shorter protein sequence compared to other PP2Cs aligned, therefore it lacks two conserved D residues required for the Mn/Mg^++^ ions interaction (Supplementary Figure S4). On the basis of protein homology and conserved functional residues we named *Capana06g000398* as a putative *CaHAI* and *Capana07g000875* as *CaABI* (circled in red, Figure 3B). Nine genes encoding *SnRK2s* were clustered in the three classical sub-families (Figure 3C, Umezawa et al., 2010), and were all already annotated in the genome. Similarly to tomato, two genes encoding subfamily III *SnRK2s* appear to be present in the genome, compared to three genes in *Arabidopsis.* Alignments of the domain II in the C-terminal region of the SnRK2 proteins separate pepper SnRK2s in SnRK2a and SnRK2b subfamilies (Supplementary Figure S5).

### *PYL*, *PP2C*, and *SnRK2* Gene Expression in Different Tissues

For gene expression analyses, we selected three *PYLs*, one for each subfamily (*CaPYL2*, *CaPYL4*, *CaPYL8*), three PP2Cs from different subclades (*CaABI*, *CaAHG1*, *CaHAI*), the two *SnRK2s* from subclass III (*CaSnRK2.3*, *CaSnRK2.6*) and one from subclass I (*CaSnRK2.4*) (Figure 3, red circles).

**FIGURE 4:**
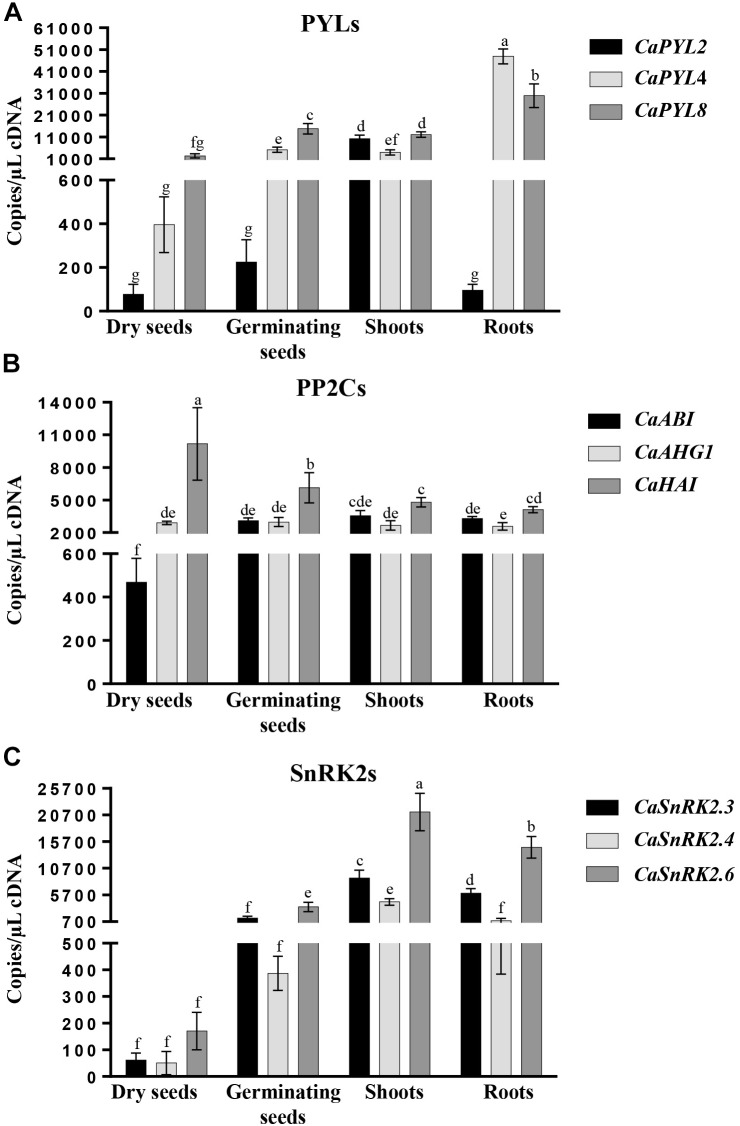
Gene expression analysis in dry seeds, germinating seeds, shoots and roots of Quadrato D’Asti giallo as monitored by absolute qRT-PCR. Expression of PYR/PYL/RCAR (*PYL*) **(A)**, *PP2C* **(B)**, and *SnRK2* **(C)** gene families. Values indicate mean ± SD (*n* = 3). Different letters indicate significant difference at *P* < 0.05 (Duncan test).

To verify organ specific expression, we performed absolute quantification through qRT-PCR in dry seeds, germinating seeds, shoots and roots (Figure 4). *CaPYL2* showed low expression in seeds, both dry and germinating, and roots, with highest levels of expression detected in shoots (about 10,000 copies/μL cDNA). *CaPYL4* had low expression in dry seeds (about 400 copies/μL cDNA), higher in germinating seeds and shoots, and the highest expression among the tested *CaPYLs* in roots (about 48,000 copies/μL cDNA) (Figure 4A). Regarding expression of *PP2Cs* in dry seeds, *CaABI* and *CaHAI* showed the lowest (about 470 copies/μL cDNA) and highest (about 10,000 copies/μL cDNA) amount, respectively (Figure 4B), while similar expression values in the other organs were detected. All three selected *SnRK2* genes showed low expression in dry seeds. *CaSnRK2.4* had low expression also in germinating seeds (around 400 copies/μL cDNA) and roots (around 800 copies/μL cDNA) while *CaSnRK2.6* showed the highest steady state level (21,000 copies/μL cDNA) in shoots (Figure 4C).

### Regulation by NaCl and ABA of *PYL*, *PP2C*, and *SnRK2* Expression

We studied the expression of selected genes in QA seeds incubated for 4 days with ABA (1, 5, 10 μM) or NaCl (25, 50, 100 mM). ABA or salt treatments resulted in up-regulation of positive ABA signaling components such as *PYLs* and *SnRK2s* (Table 1). In particular, *CaPYL4* expression was significantly up-regulated by the highest concentration of ABA, whereas *CaPYL8* was upregulated by all NaCl treatments. *CaPYL2* expression level was below detection. *CaSnRK2.6* was up-regulated in all tested treatments except for the lowest ABA concentration, while *CaSnRK2.3* was induced by ABA 10 μM, NaCl 50 and 100 mM. *CaSnRK2.4* was up-regulated by ABA 10 μM and all NaCl treatments. *CaHAI* was the only PP2C gene up-regulated by ABA and all NaCl treatments while *CaAHG1* was up-regulated only by ABA 1 μM. We also tested shoot and root expression of QA seedlings grown in hydroponic solution and subjected to 3 h salt stress treatments (Table 2). In this case, the three tested PP2C genes were significantly up-regulated in both tissues. By contrast, positive regulators were less responsive; *CaSnRK2.6* was the only *SnRK2* up-regulated in shoot. Notably, *CaPYL4* was strongly down-regulated after NaCl treatment in both shoots and roots.

**Table 1:** Relative quantification of gene expression measured by qRT-PCR of selected genes in seeds of Quadrato D’Asti giallo incubated for 4 days on control medium and media with different concentrations of ABA or NaCl.

	Control	ABA 1 μM	ABA 5 μM	ABA 10 μM	NaCI 25 mM	NaCI 50 mM	NaCI 100 mM
*CaPYL4*	1	1.561 ± 0.014	1.787 ± 0.005	1.855 ± 0.005*	1.267 ± 0.004	1.558 ± 0.005	1.603 ± 0.005
*CaPYL8*	1	0.850 ± 0.030	1.093 ± 0.014	1.492 ± 0.027	2.278 ± 0.013***	2.216 ± 0.011***	1.778 ± 0.007***
*CaABI*	1	0.751 ± 0.001	0.756 ± 0.001	1.177 ± 0.001	0.873 ± 0.001	0.960 ± 0.001	1.282 ± 0.001
*CaAHGl*	1	1.652 ± 0.009*	0.770 ± 0.005	0.827 ± 0.003	0.866 ± 0.001	0.760 ± 0.001	0.990 ± 0.001
*CaHAI*	1	0.331 ± 0.001*	0.592 ± 0.003	3.142 ± 0.004***	2.546 ± 0.002*	3.786 ± 0.005*	4.567 ± 0.004***
*CaSnRK2.3*	1	0.851 ± 0.007	0.844 ± 0.017	1.486 ± 0.018*	1.071 ± 0.010	1.321 ± 0.009**	1.340 ± 0.014*
*CaSnRK2.4*	1	0.921 ± 0.009	1.401 ± 0.006	1.870 ± 0.010*	2.085 ± 0.011*	1.785 ± 0.008*	2.176 ± 0.010**
*CaSnRK2.6*	1	1.371 ± 0.003	3.269 ± 0.004**	3.706 ± 0.003***	3.169 ± 0.003**	2.971 ± 0.002***	5.211 ± 0.006***


*All data are expressed relative to control treatment as mean ± SD (n = 4). Asterisks represent significance levels using Student t-test; ^∗^P ≤ 0.05; ^∗∗^P ≤ 0.01; ^∗∗∗^P ≤ 0.005.*

**Table 2:** Relative quantification of gene expression measured by qRT-PCR of selected genes in roots and shoots of Quadrato D’Asti giallo seedlings grown in hydroponic system after 3 h of 200 mM NaCl treatment.

	Control	NaCI 200 mM	
		Shoots	Roots
*CaPYL2*	1	0.887 ± 0.001	*B.D.*
*CaPYL4*	1	0.288 ± 0.001*	0.217 ± 0.004***
*CaPYL8*	1	0.992 ± 0.002	1.352 ± 0.067
*CaABI*	1	2.400 ± 0.002*	2.756 ± 0.001***
*CaAHGl*	1	1.915 ± 0.045*	1.993 ± 0.001*
*CaHAI*	1	32.662 ± 0.027***	17.009 ± 0.007***
*CaSnRK2.3*	1	1.247 ± 0.001	0.864 ± 0.009
*CaSnRK2.4*	1	1.072 ± 0.002	0.455 ± 0.006
*CaSnRK2.6*	1	1.821 ± 0.004*	1.067 ± 0.010


*All data are expressed relative to control treatment as mean ± SD (n = 3). Asterisks represent significance levels using Student t-test; ^∗^P ≤ 0.05; ^∗∗^P ≤ 0.01; ^∗∗∗^P ≤ 0.005. B.D., below detection.*

### Interaction Between *PYLs*, *PP2Cs*, and *SnRK2s*

**FIGURE 5:**
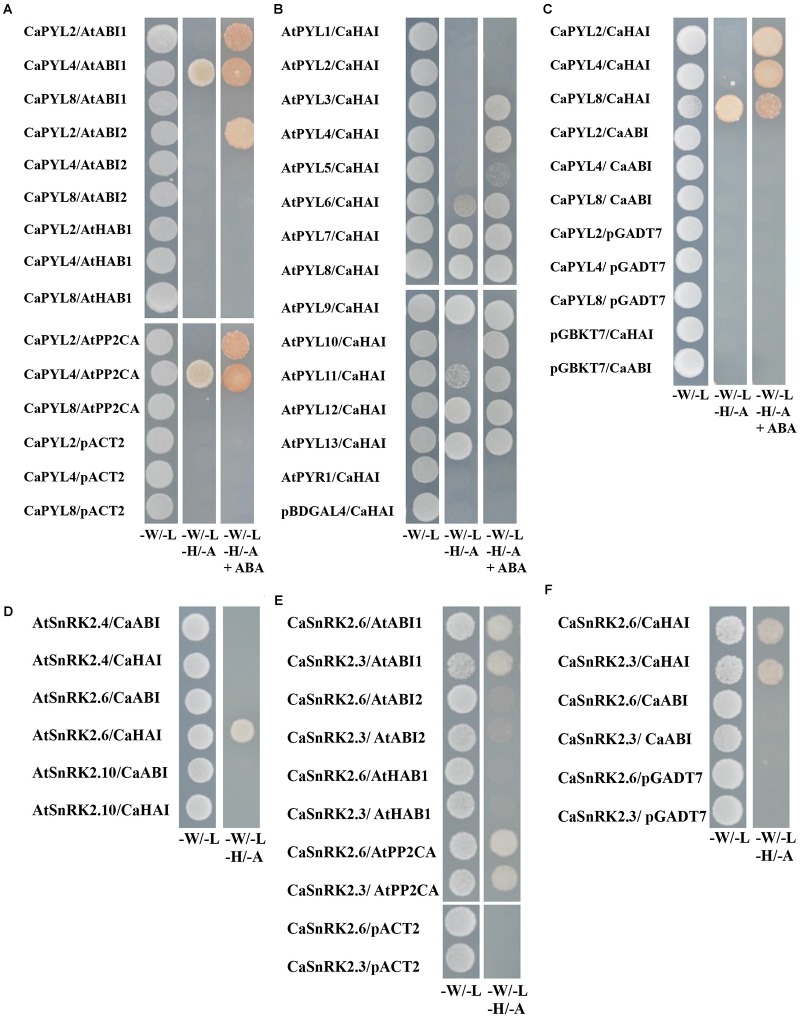
Interaction test of PP2Cs with PYLs and SnRK2s in the yeast two-hybrid assay. **(A)** AtPP2Cs fused to the GAL4 Activation Domain (AD) were co-transformed in yeast with CaPYLs cloned in frame with the GAL4 binding domain (BD) of pGBKT7, with combinations shown in figure. **(B)** CaHAI fused to the AD in pGADT7 was expressed in yeast with AtPYLs in pBDGAL4, the different combinations are shown. **(C)** CaPP2Cs were fused to the AD in pGADT7 and co-transformed with CaPYLs cloned in pGBKT7. **(D)** CaPP2Cs in pGADT7 were co-transformed in combination with AtSnRK2s cloned in pGBKT7. **(E)** AtPP2Cs in pACT2 were co-transformed in yeast with CaSnRK2s in pGBKT7, with combinations shown in figure. **(F)** Co-transformants of yeast containing CaPP2Cs fused to the AD in pGADT7 in combination with CaSnRK2s in pGBKT7. Co-transformations of the pepper PYL/SnRK2/PP2C orthologs with appropriate complementary empty vectors are shown as negative controls. Yeast cells grown on synthetic media (–W/–L) and on synthetic, selective media without (–W/–L/–H/–A) or, where indicated, with 50 μM ABA (–W/–L/–H/–A+ABA) are shown. Pictures were taken after 3 days of incubation at 30°C.

**FIGURE 6:**
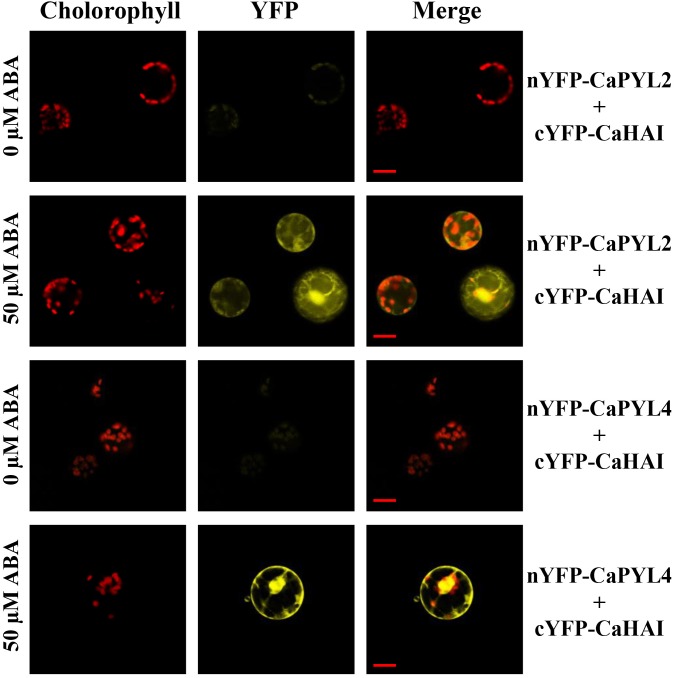
BiFC assay in *Nicotiana tabacum* protoplasts. CaHAI fused with the C-terminus of YFP (cYFP) was cotransformed into protoplasts, respectively, with CaPYL2 and CaPYL4 fused with N-terminus of YFP. Chlorophyll autofluorescence, YFP fluorescence and merged images are shown in the presence or absence of ABA. Scale bars = 20 μm in all panels.

The key function of ABA receptors upon ABA binding is their ability to interact with and inhibit PP2Cs, releasing SnRK2s from inhibition (Fujii et al., 2009; Park et al., 2009). We therefore tested whether selected CaPYLs, CaPP2Cs and CaSnRK2s were capable of interaction with each other or with *Arabidopsis* members of the ABA signaling cascade in the yeast two hybrid assay. *CaPYLs* and *CaSnRK2s* were fused to the GAL4 Binding Domain (BD) and the PP2Cs *CaABI* and *CaHAI* to the GAL4 activation domain (AD) and used to test multiple combinations (Figure 5). CaPYL4 interacted with AtABI1 and AtPP2CA irrespective of the presence of ABA, while CaPYL2 interaction with AtABI1, AtABI2 and AtPP2CA was only observed in presence of the hormone (Figure 5A). The ability of CaHAI to interact with AtPYLs in presence or not of ABA was also tested (Figure 5B). Yeast co-transformed with CaHAI and, respectively, AtPYL6, AtPYL7, AtPYL8, AtPYL9, AtPYL11, AtPYL12 and AtPYL13 was able to grow on selective media (–W/–L/–H/–A). Interactions between AtPYL6, AtPYL11 and CaHAI were weak without ABA, but increased markedly when ABA was present. Furthermore on selective media containing ABA, the yeast co-transformed with AtPYL3/CaHAI, AtPYL4/CaHAI and AtPYL10/CaHAI combinations was able to grow in addition to other combinations mentioned above. The combination AtPYL5/CaHAI showed a weak growth on the selective media with ABA (Figure 5B). No interaction was observed for any transformation with the cloned CaABI and each of the AtPYLs (Supplementary Figure S6) or CaPYL2/4/8 (Figure 5C). In the test between CaPYLs and CaHAI, we observed interaction in presence of ABA with CaPYL2/4/8, while without ABA only the combination CaPYL8/CaHAI showed growth (Figure 5C). The interactions between PP2Cs and SnRK2s of *C. annuum* and *Arabidopsis* were also analyzed in yeast (Figure 5D–F). When we tested combinations between AtSnRK2s and CaPP2Cs, only the yeast co-transformed with the member of subfamily III AtSnRK2.6 and CaHAI was able to grow on the selective media (Figure 5D). Similarly, we observed interaction between CaSnRK2.3/2.6 and AtABI1 and AtPP2CA (Figure 5E). A positive interaction between CaPP2Cs and CaSnRK2s was observed when yeast was co-transformed with CaHAI and CaSnRK2.3 or CaSnRK2.6, while the cloned CaABI was incapable of binding to SnRK2s in yeast (Figure 5F). CaABI did not interact with any of the *Arabidopsis* or pepper ABA receptors and SnRK2s in yeast (Figure 5C,D,F and Supplementary Figure S6), although the protein was produced in yeast, as verified through western-blot by the presence of a signal of the expected molecular weight (Supplementary Figure S7). We confirmed in a plant system the interaction between CaHAI and CaPYL2/4 in presence of ABA. We used a split-reporter system in tobacco protoplasts by transient co-expression of CaHAI and CaPYL2/4 fused to complementary fragments of the YFP. A reconstituted fluorescence signal was observed for both interactions only in presence of ABA (50 μM, Figure 6), thus confirming the results obtained in yeast. The interaction CaHAI-CaPYL2 was observed within 5 min of incubation with ABA, while a 2 h incubation was allowed to detect the CaHAI-CaPYL4. All negative controls with or without ABA are reported in Supplementary Figure S8.

We also investigated the subcellular localization of the proteins CaABI, CaHAI, CaPYL2, and CaPYL4 (Supplementary Figure S9). CaABI, CaPYL2, and CaPYL4 showed a nuclear and cytoplasmic localization, while for YFP-CaHAI the fluorescence signal was present only in the nucleus (Supplementary Figure S9).

## Discussion

Seed germination and seedling growth are adversely affected by ABA and NaCl. *Capsicum annuum* is classified as a moderately salt-sensitive species (De Pascale et al., 2003; Aktas et al., 2006; Penella et al., 2016). Here, we have shown that pepper cv. Quadrato D’Asti giallo (QA) had reduced seed germination and a complete inhibition of cotyledon expansion at 10 μM ABA. Similarly, the presence of 100 mM NaCl caused a 1 day delay in seed germination and reduced cotyledon expansion. Therefore, QA seeds displayed an inhibited germination only at the highest NaCl or ABA concentrations applied (Figure 1). At seedling stage, we observed a reduction of root length and shoot weight in seedlings grown in the presence of 100 mM NaCl (Figure 2), consistent with previous reports of a reduced vegetative growth in presence of NaCl (Yildirim and Güvenç, 2006).

NaCl-induced inhibition of germination in pepper is mainly due to the osmotic component of salt stress (Chartzoulakis and Klapaki, 2000; Demir and Mavi, 2008), which is counteracted in plants by employing ABA-dependent mechanisms (Cutler et al., 2010; Nakashima and Yamaguchi-Shinozaki, 2013).

Differences in sensitivity to ABA/NaCl between developmental stages/tissues, which may also contribute to explain the known phenomenon of pepper vivipary and low seed longevity, might be at least partially due to the expression/activity of ABA signaling components. Therefore, through a sequence similarity search, we identified putative pepper orthologs of *PYLs*, clade A *PP2Cs* and *SnRK2s*. Reflecting a high degree of conservation of these families among land plants, cluster analysis showed the classical organization in 3 subfamilies for pepper *PYLs* and *SnRK2s*. However, similarly to the tomato *PYLs*, no close relative for the *Arabidopsis* subgroup *AtPYL11/12/13* was found (Gonzalez-Guzman et al., 2014). *Capana02g001761*, annotated as *CaPYL12-like*, did not cluster in any of the classical three subfamilies (Figure 3A), and was in a clade with the putative tomato ortholog *Solyc02g076770*, also ungrouped in the study of Gonzalez-Guzman et al. (2014). Capana02g001761, as its tomato ortholog, lacked key aminoacids, including residues of conserved loop 2 (CL2), CL3, and CL4 (Supplementary Figure S2). In contrast, presence of these conserved residues, as well as the ABA-dependent interaction with PP2C observed in yeast and protoplasts suggests that CaPYL2 and CaPYL4 are functional ABA receptors.

Additionally, the functional residues or domains were well conserved in the deduced sequences of three selected CaPP2Cs (Supplementary Figures S3, S4). Similarly to *Arabidopsis* AHG1 and Solyc03g006960, CaAHG1 lacks the tryptophan (W385 in HAB1, W300 in ABI1) that in other clade A PP2Cs participates to the binding with ABA (Weiner et al., 2010).

The domain II in the C-terminal region of the SnRK2 proteins is critical for PP2C interaction and ABA responsiveness (Kobayashi et al., 2005; Yoshida et al., 2006). Seven pepper SnRK2s (Capana06g001594, Capana01g000732, Capana08g002441, Capana02g003135, Capana05g000287, Capana12g002655, Capana04g000996) belong to subgroup 3 and 2, which in *Arabidopsis* can be strongly or weakly activated by ABA; while Capana08g001898 and Capana00g002391 cluster with subgroup 1, containing SnRK2s that may not be activated by ABA treatment (Figure 3C and Supplementary Figure S5; Kulik et al., 2011).

Quantification of gene expression of selected genes in different organs showed differences among family members (Figure 4).

*CaPYL2* was highly expressed in shoots, while *CaPYL4* and *CaPYL8* showed highest expression in roots. These data are consistent with previous studies in tomato (Gonzalez-Guzman et al., 2014) and soybean (Bai et al., 2013). Interestingly, differences in transcript levels of the tested PP2Cs were observed only in seeds. *CaHAI* had highest steady state expression levels in dry seeds. By contrast, *CaABI* had the lowest expression in dry or germinating seeds and similar expression in all vegetative tissues. Finally, SnRK2 kinases, including the two subclass III members, also had lowest expression in dry seeds. In other species, SnRK2s were shown to be highly expressed in seeds (Fujii et al., 2007; Nakashima et al., 2009; Lou et al., 2017). Considering the established role of these kinases in plant responses to abiotic stress (Zhu, 2016), the low *SnRK2* expression in seed suggests an ineffective mechanism of ABA signaling that may affect sensitivity to ABA at the germination stage. However, it is possible that other *SnRK2s* genes, not tested here, might be highly expressed in seeds. In addition, activity of ABA signaling components is regulated post-translationally through ABA binding, protein interaction and/or phosphorylation/dephosphorylation. Thus, the gene expression in specific organs or in response to treatments only provides an initial indication of the effectiveness of this mechanism, which will have to be verified by measurements of protein activity.

In seeds, 10 μM ABA treatment induced up-regulation of PP2C *CaHAI*, indicating a possible negative feedback at high ABA concentrations, as well as of all the tested *SnRK2s*. ABA-induced up-regulation of *SnRK2s* has been reported in the case of vegetative tissues, and is thought to derive from a self-amplification mechanism of ABA signal transduction components (Wang et al., 2018). Interestingly, 10 μM ABA treatment also induced *CaPYL4* expression, while in vegetative tissues PYLs are commonly down-regulated by ABA (Wang et al., 2018).

In seedlings, *CaPYL4* was significantly down-regulated after NaCl treatment in shoots and roots, while *CaPYL8* did not change significantly (Table 2). Similarly, in tomato seedlings exposure to dehydration caused repression of *SlPYL4* expression, while *SlPYL8* showed no change in gene expression (Sun et al., 2011). By contrast, all genes within the *SlPP2C* gene family were significantly up-regulated by dehydration (Sun et al., 2011). In rice, expression of 10 *OsPP2C* genes in plants treated with 150 mM NaCl was up-regulated (Xue et al., 2008). Consistently, we observed NaCl-induced up-regulation for *CaABI*, *CaHAI*, and *CaAHG1* (Table 2).

Protein-protein interaction assays through Y2H between pepper core signaling components or with Arabidopsis orthologs showed ABA-dependent and -independent interactions (Figure 5), as observed in examples from other crops (Wang et al., 2018). CaHAI showed an interaction pattern similar to the *Arabidopsis* orthologs (Bhaskara et al., 2012). In BiFC, interaction between CaHAI and CaPYL2 or CaPYL4 were both ABA-dependent and localized to the nucleus and cytoplasm, indicating that both transcriptional and immediate responses to ABA can be regulated by these complexes (Figure 6).

Although all the data here gathered points toward CaPYL2/4, CaHAI, and SnRK2.3/2.6 as ABA receptors, PP2C phosphatases and SnRK kinases, respectively, *in vitro* activity assays are needed to truly probe this point.

Notably, a CaABI transcript, annotated as splicing isoform 4 (XM_016724784.1), amplified from dried/germinating seeds, encoded a truncated protein lacking two conserved D residues required for the Mn/Mg^++^ ions interaction. When used for interaction assays was not capable to complex with PYLs or SnRK2s, suggesting that this variant may not be functional. Alternative splicing of pre-mRNAs constitutes an additional layer of regulation of gene expression and a way to expand the proteome diversity or regulate protein functionality in specific developmental/environmental situations, or cell types (Laloum et al., 2018). In plants, intron retention is indicated as the most common alternative splicing type (Ner-Gaon et al., 2004). Stress and ABA-response regulatory genes are known to be most prone to alternative splicing phenomena, which may produce active vs. inactive protein variants (Laloum et al., 2018). Specifically, splicing variants have been described in the case of the *Arabidopsis* *HAB1* PP2C (Ling et al., 2017). The ratio between the full-length, functional HAB1 protein and the truncated splice form resulting from intron retention are regulated by the ABA levels and are involved in the fine-tuning of the ABA signaling cascade (Wang et al., 2015). The splicing variant of CaABI expressed in seeds may result from a similar mechanism of balance of phosphatase activity, and may reflect a developmental rather than environmental regulation between protein isoforms.

## Conclusion

In conclusion, pepper seeds retained high germination percentages in presence of NaCl and ABA. This may be due to low expression in seeds of ABA signaling components such as *CaABI*, *CaPYL2*, *CaPYL4*, *CaSnRK2.3, CaSnRK2.6*. The expression of tested *PYLs* and *SnRK2s* was comparatively higher in vegetative tissues, and this correlated with restored sensitivity to exogenous ABA and salinity. Expression of a splice variant of *CaABI* encoding a truncated protein unable to interact with other tested signaling components may also impair ABA signaling cascade in seeds. The alleles controlling the expression of these signaling components in maturing and dry seed are potential traits that can be manipulated for the purposes of improving pepper low seed storability through molecular breeding and gene editing.

## Data Availability

All datasets generated for this study are included in the manuscript and/or the Supplementary Files.

## Author Contributions

AR, SG, and GB designed the work. AR, SL, PP, and MP performed the experiments and prepared the figures. AR, SL, MVO, and AC analyzed the data. GM and AM participated in the experimental design and data interpretation. AR, MVO, and GB wrote the manuscript, with inputs from all authors. All the authors have read and approved the manuscript.

## Conflict of Interest Statement

The authors declare that the research was conducted in the absence of any commercial or financial relationships that could be construed as a potential conflict of interest.

## Footnotes

1 www.fao.org

2 https://www.genome.jp/tools-bin/clustalw

3 http://www.ch.embnet.org/software/BOX_form.html
