# Supplementary material for: Salinity and ABA Seed Responses in Pepper: Expression and Interaction of ABA Core Signaling Components
Source: Front Plant Sci. 2019 Mar 19;10:304. doi: 10.3389/fpls.2019.00304 (PMC6433719; doi:10.3389/fpls.2019.00304)
Supplement: Supplementary file 1 [file Data_Sheet_1.PDF]

Supplementary Data for the Manuscript SALINITY AND ABA SEED RESPONSES IN PEPPER: EXPRESSION AND INTERACTION OF ABA CORE SIGNALING COMPONENTS (Ruggiero et al.)

Supplementary Materials and Methods

Yeast protein extraction and western blot analysis

Yeast protein was extracted using 0.2 M NaOH (Kushnirov, 2000). Cells were grown overnight in liquid culture lacking leucine and tryptophan (-W/-L). The liquid culture was centrifuged 5 minutes at 4000 rpm and the proteins were extracted using 0.2 M NaOH. The extracts were centrifuged again, resuspended in SDS buffer and resolved by SDS PAGE. After blotting, membrane was incubated in blocking buffer (TBS containing 0.1% v/v Tween and 5% w/v BSA), for 1 h at room temperature and challenged with primary anti-HA antibody (Thermo Fisher Scientific, USA), diluted (1:20000 dilution) in TBS containing 0.1% v/v Tween and 1% w/v BSA, and incubated 1 h at room temperature. A HRP-conjugated anti-mouse antibody (GE Healthcare) was applied for 1 h at room temperature. Detection was carried out with ECL (GE Healthcare) following manufacturer’s instructions.

Supplementary References

Kushnirov, V. V. (2000). Rapid and reliable protein extraction from yeast. *Yeast*, *16*(9), 857-860. https://doi.org/10.1002/1097-0061(20000630)16:9<857::AID-YEA561>3.0.CO;2-B

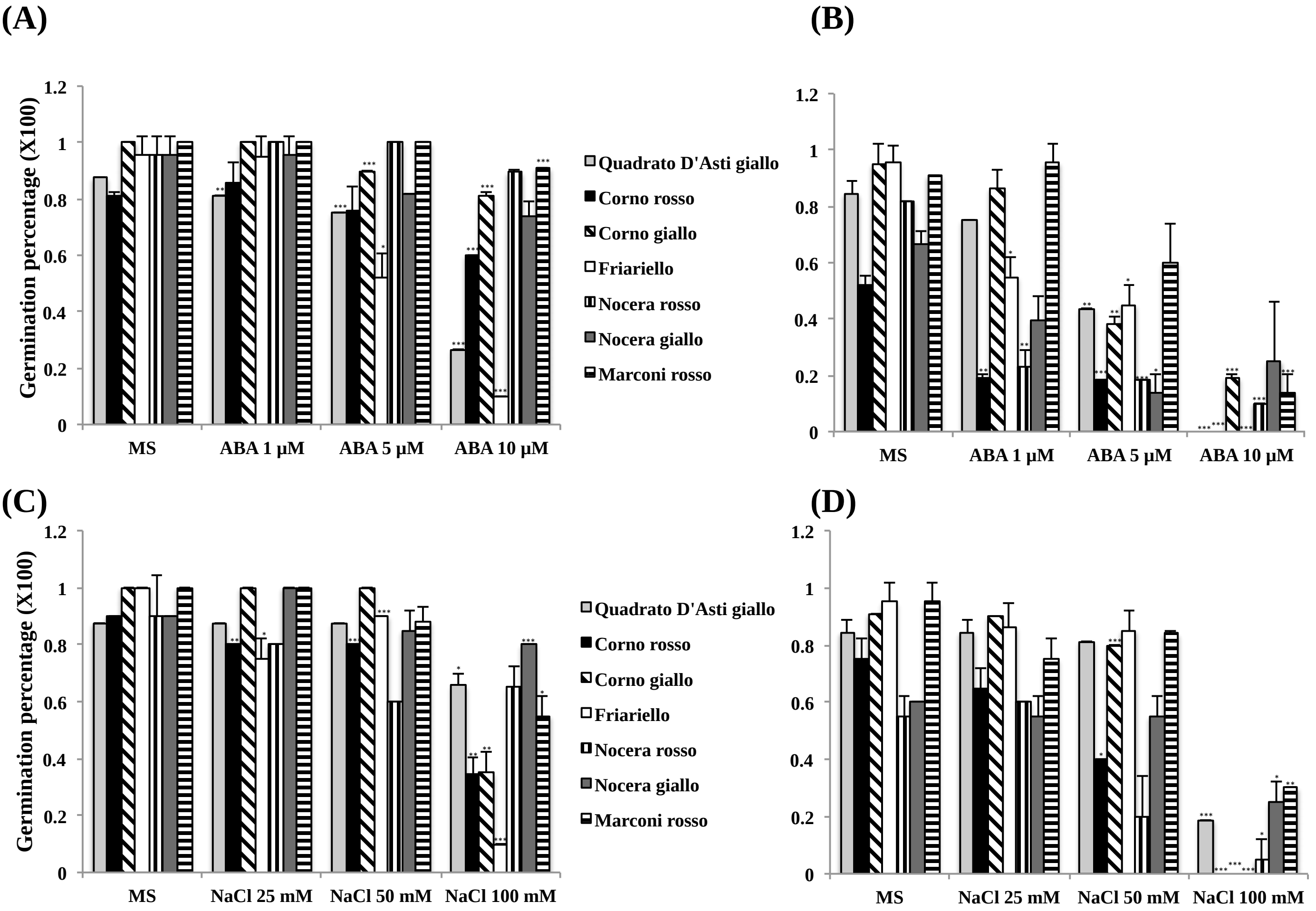

Figure S1: Germination percentage in terms of radicle emergence (day 6) (**A**,**C**) and cotyledon expansion (day 11) (**B**,**D**) of seeds treated with different concentrations of ABA or NaCl. Values indicate mean ± SD (n=20). Asterisks represent significance levels using Student t-test; * P value ≤ 0.05; ** P value ≤ 0.01; *** P value ≤ 0.005.

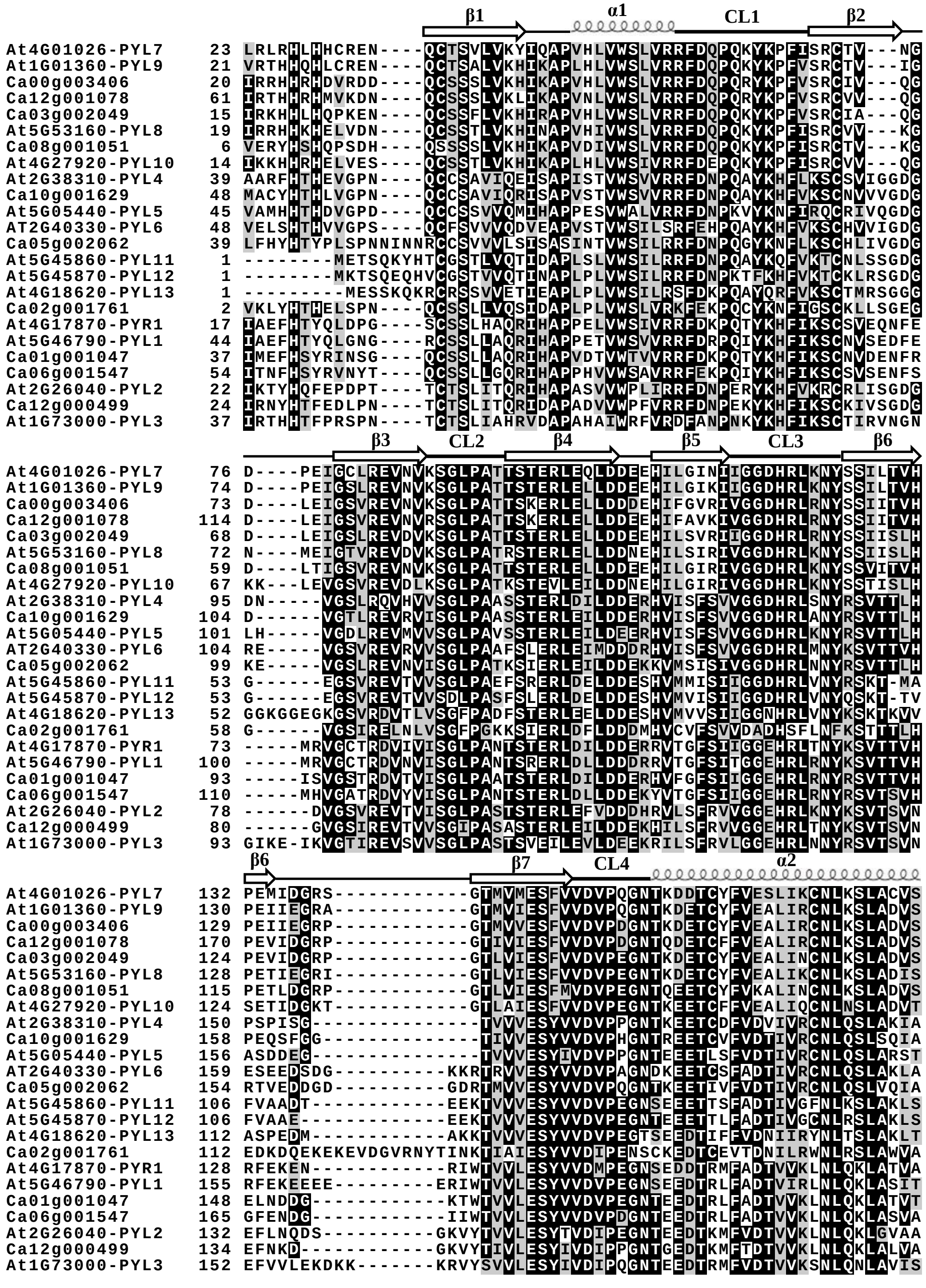

Figure S2: Aminoacid sequence alignment of *A. thaliana* and *C. annuum* PYLs. Secondary structural elements are indicated above the sequence alignment. The four conserved loops CL1-CL4 are highlighted by thick lines. Aminoacid sequences are shown starting from the indicated aminoacid position. Conserved residues are marked with black, while grey shading indicates residues with similar properties.

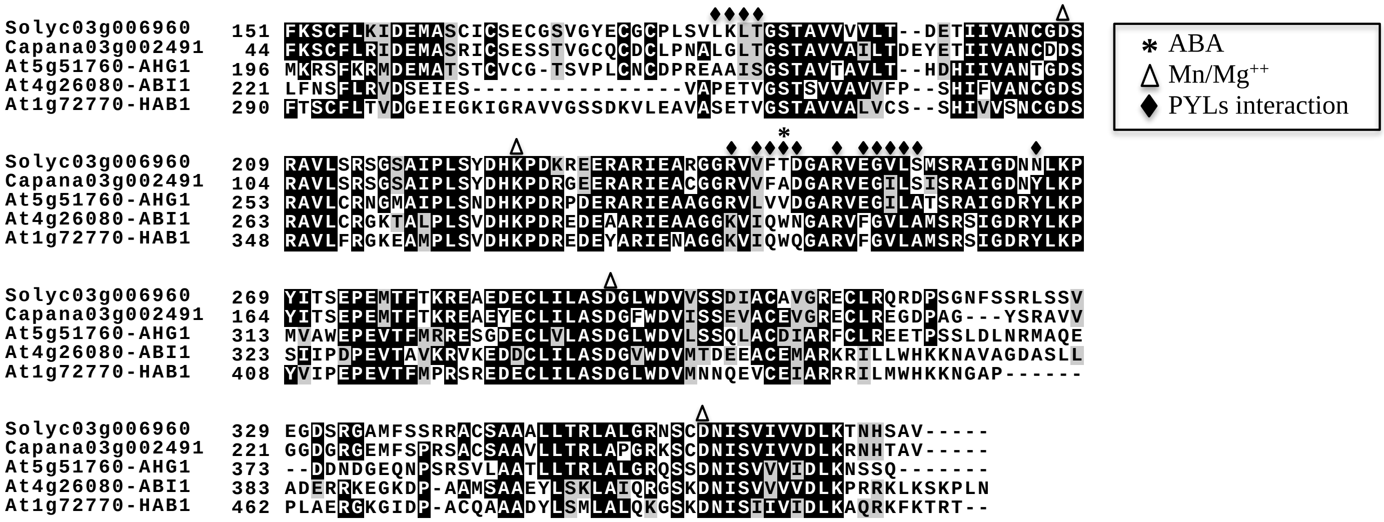

Figure S3: Aminoacid sequence alignment of the cloned CaPP2C (Capana03g002491) and its closest *Arabidopsis thaliana* and *Solanum lycopersicum* orthologs. Aminoacid sequences are shown starting from the indicated aminoacid position. Conserved residues are marked with black, while grey shading indicates residues with similar properties. Residues interacting with ABA, Mn/Mg ions and PYLs are marked with asterisks, white triangles and black diamonds, respectively.

Functional residues are based on studies by Melcher et al., 2010 and Santiago et al., 2012.

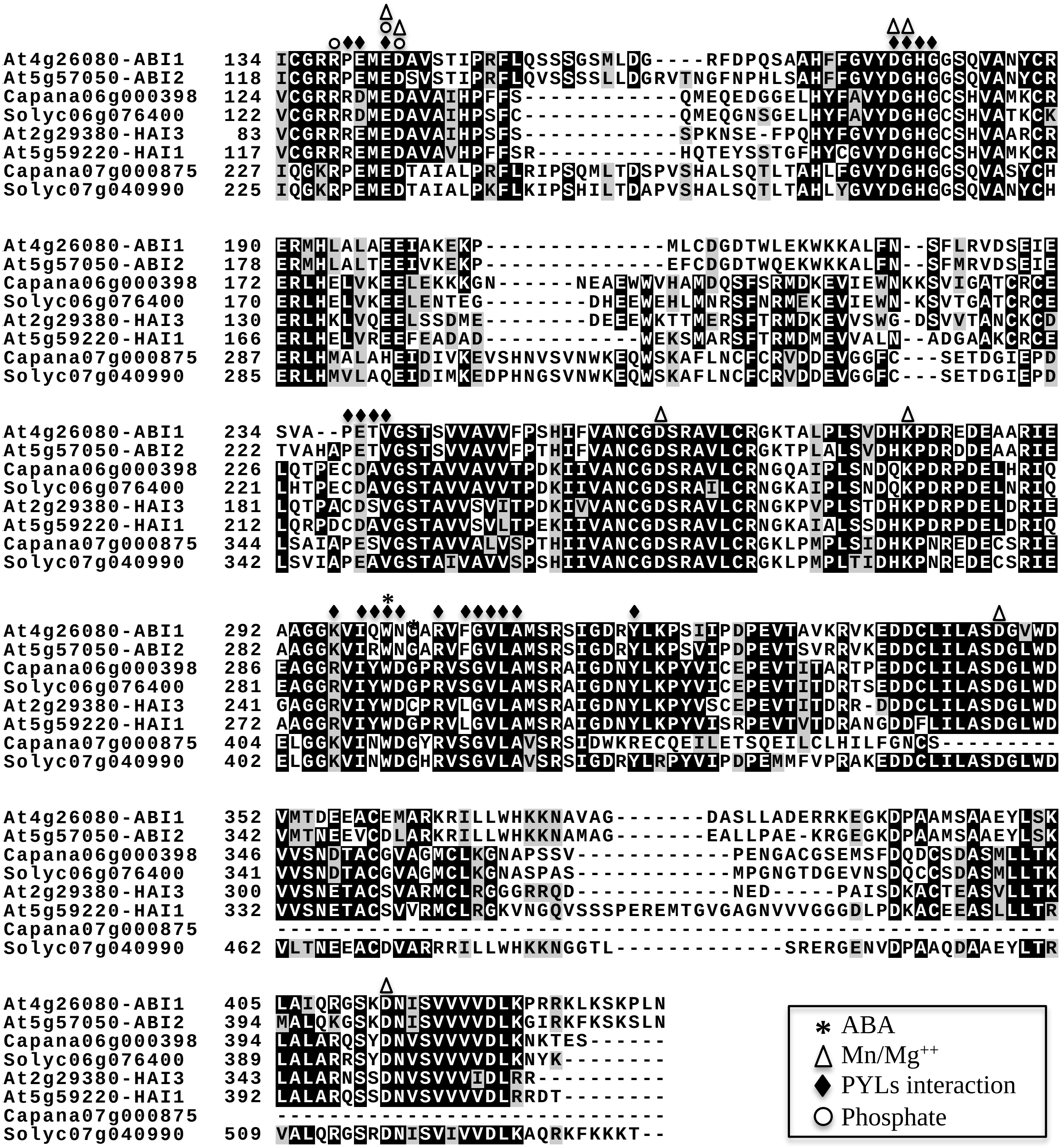

Figure S4: Aminoacid sequence alignment of Capana06g000398, Capana07g000875 cloned from pepper dry seeds and their closest *Arabidopsis thaliana* and *Solanum lycopersicum* orthologs. Aminoacid sequences are shown starting from the indicated aminoacid position. Conserved residues are marked with black, while grey shading indicates residues with similar properties. Residues interacting with ABA, Mn/Mg ions and PYLs are marked with asterisks, white triangles and black diamonds, respectively. Phosphatase sites are marked with white circles.

Functional residues are based on studies by Melcher et al., 2010 and Santiago et al., 2012.

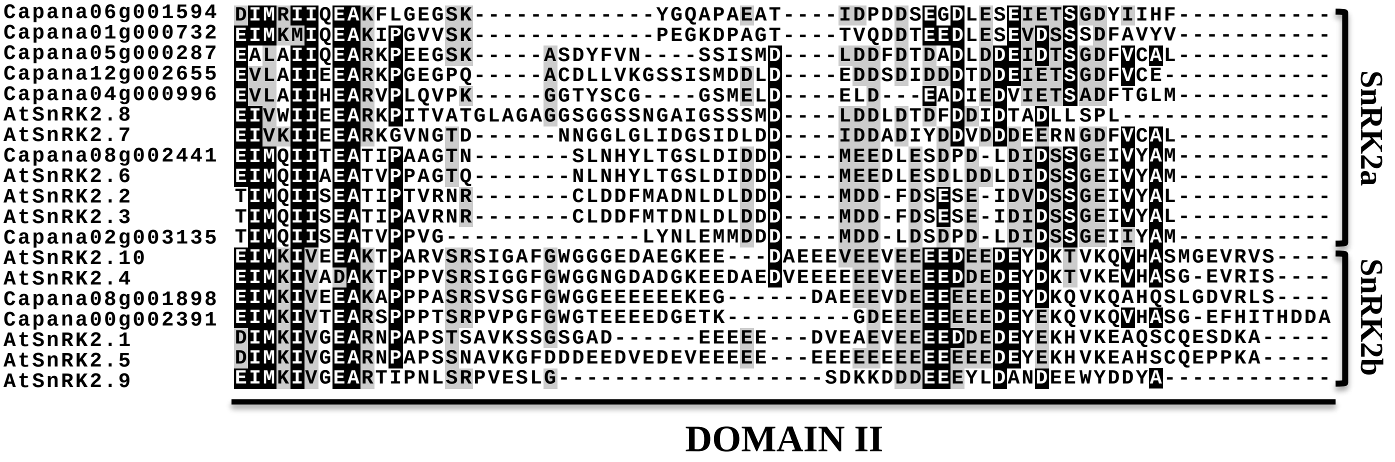

Figure S5: Aminoacid sequence alignment of the C-terminal region in *A. thaliana* and *C. annuum* SnRK2s. According to the alignment of the domain II in the C-terminal region, SnRK2s are classified into SnRK2a and SnRK2b. Conserved residues are marked with black, while grey shading indicates residues with similar properties.

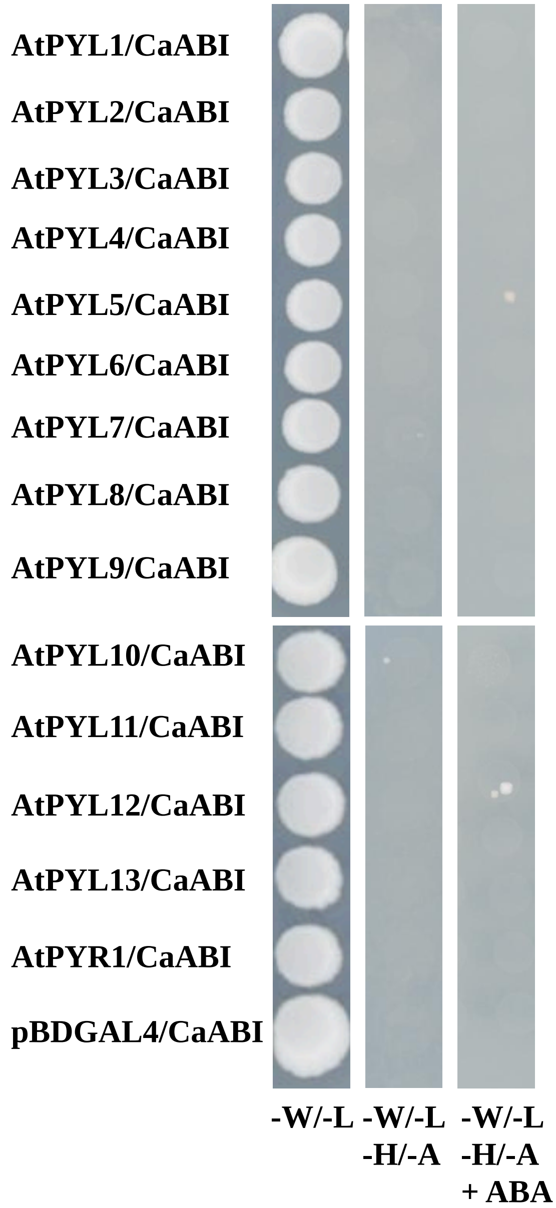

Figure S6: Interaction test of CaABI with AtPYLs in the yeast two-hybrid assay. CaABI fused to the GAL4 Activation Domain (AD) was co-transformed in yeast with AtPYLs cloned in frame with the GAL4 binding domain (BD) of pBDGAL4, with combinations shown in figure. Yeast cells grown on synthetic media (–W/–L) and on synthetic, selective media without (–W/–L/–H/–A) or with 50 µM ABA (–W/–L/–H/–A+ABA) are shown. Pictures were taken after 3 days of incubation at 30°C.

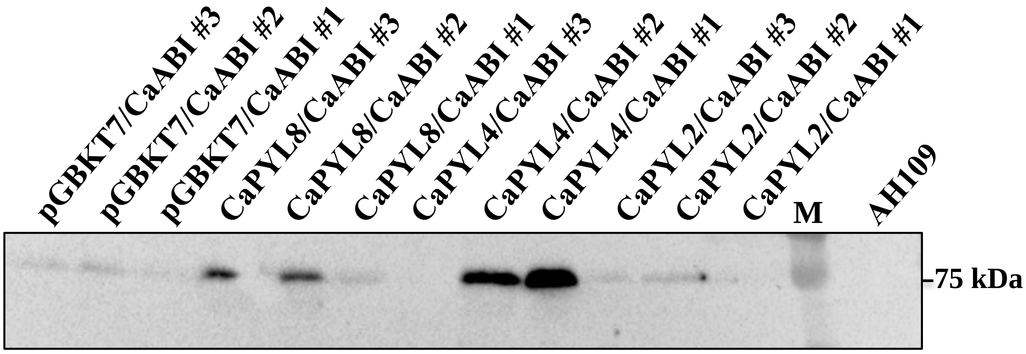

Figure S7: Western blot of protein extracts of yeast colonies co-transformed with the indicated combinations. Anti-HA antibody was used to detect signal, as detailed in supplementary material and methods. Three different colonies (#1-3) per combination were tested; M, marker; AH109, untransformed yeast strain, used as negative control.

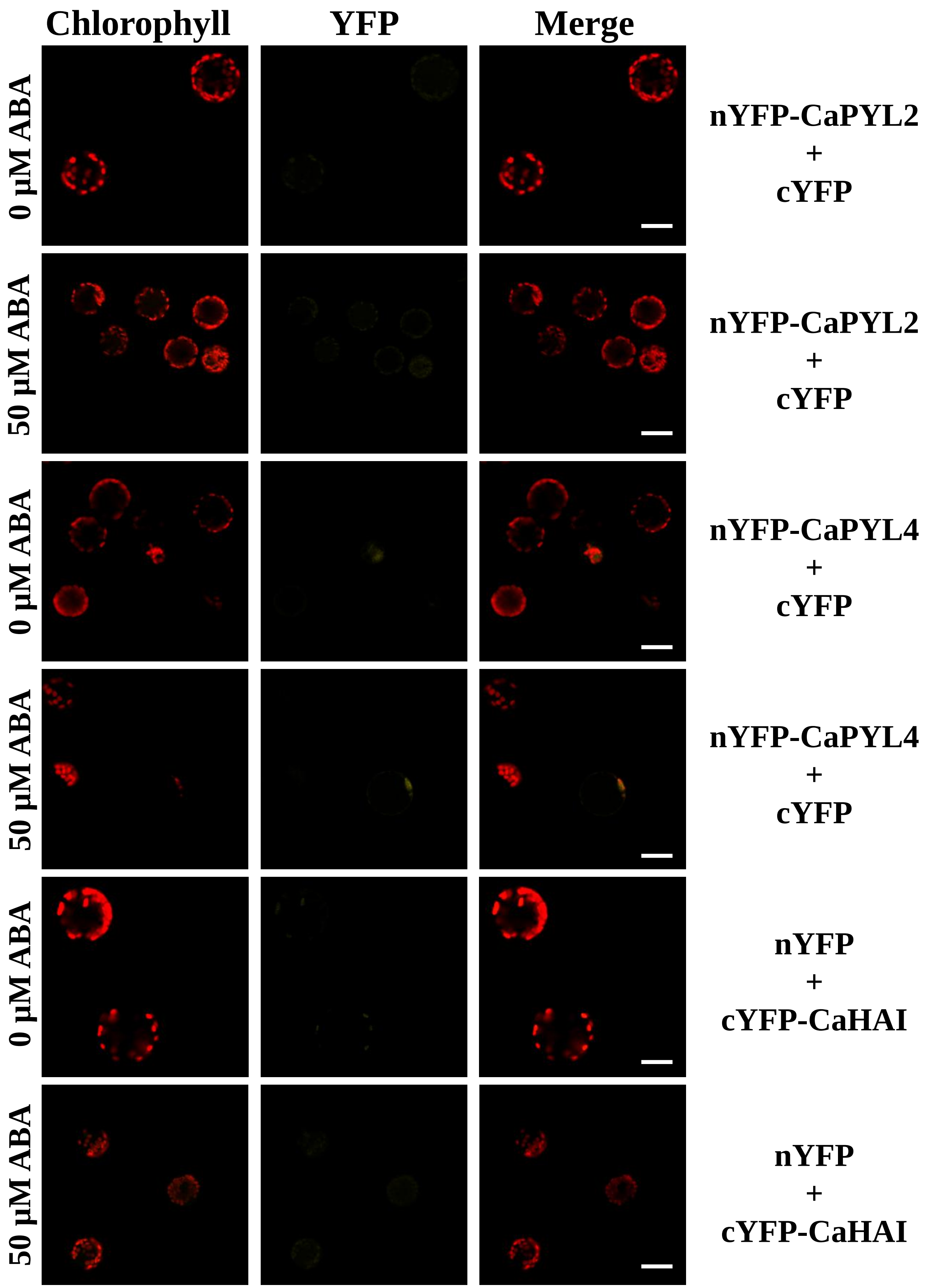

Figure S8: BiFC assay in *Nicotiana tabacum* protoplasts. Combinations tested are shown in figure. Images were taken from samples untreated or treated for 2h with 50 µM ABA. Chlorophyll autofluorescence, YFP fluorescence and merged images are shown. Scale bars = 20 µm in all panels.

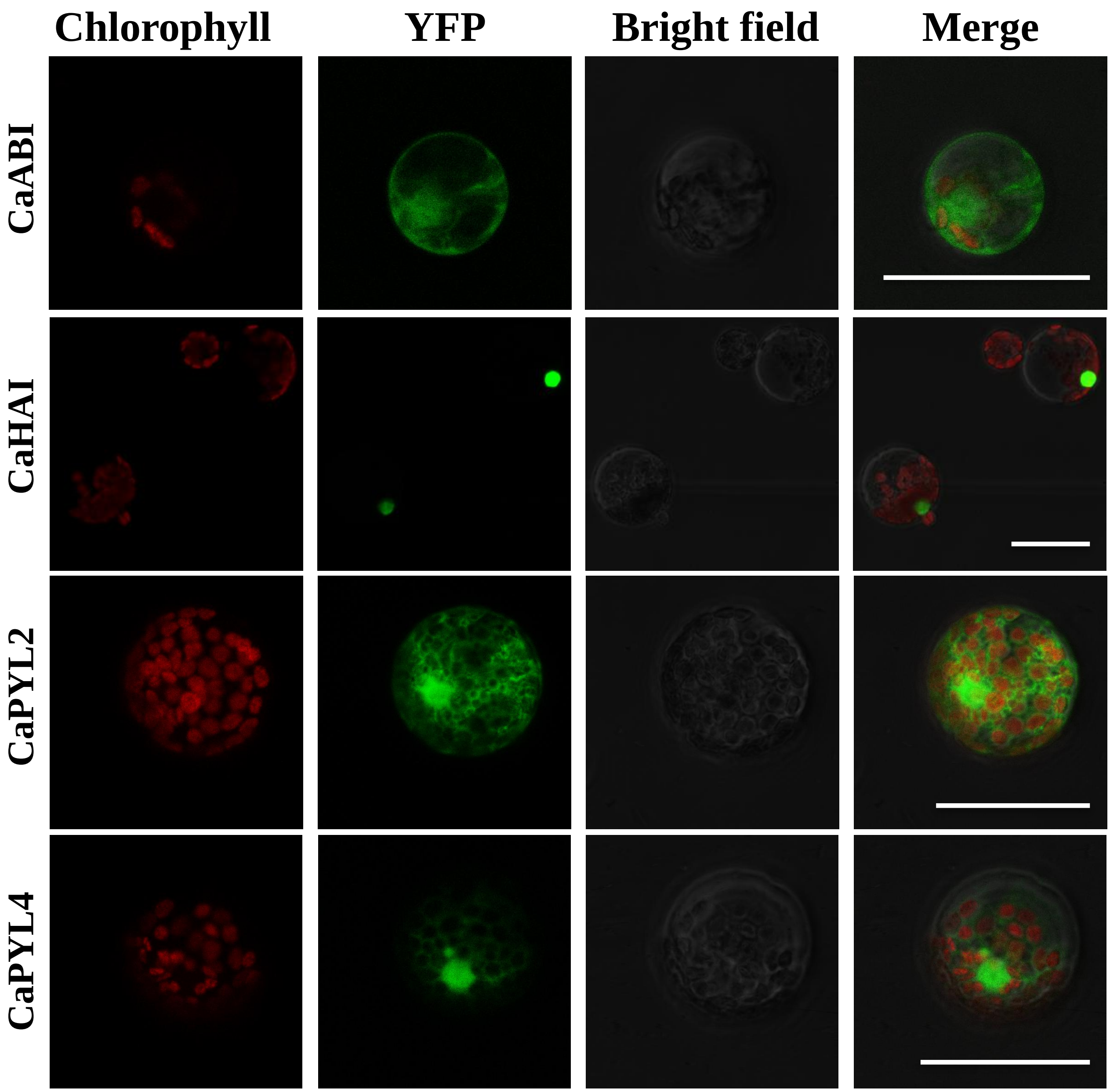

Figure S9: Localization of YFP-CaABI/HAI/PYL2/PYL4 expressed in *Nicotiana tabacum* protoplasts. YFP fluorescence, chlorophyll autofluorescence, bright field and merged images are shown. Scale bars = 50 µm in all panels.

Table S1. Primers used for absolute and relative qRT-PCR

Name	Sequence (5'-3')
CaABI For	ACCCCGAGCAAAAGAAGACG
CaABI Rev	CCGTGCTACATCACAGGCTT
CaAHG1 For	CCGGAGATGACCTTCACCAA
CaAHG1 Rev	CCTCACATGCTACCTCGCTT
CaEIF5A2 For	CCTGTTATCGTGCTACTTTG
CaEIF5A2 Rev	GTTTCATTGCCNTGCCAGAT
CaHAI For	CGATCAAAAGCCGGATCGTC
CaHAI Rev	GAACACCTGAAACACGAGGG
CaPYL2 For	CCACCAGGGAATACTGGTGAA
CaPYL2 Rev	TGCAGACATTGCCACTAAAGC
CaPYL4 For	ACCACACTCACCTTGTTGGT
CaPYL4 Rev	CGAACCACGGACCATACTGT
CaPYL8 For	CCTGCAACAACGAGTACCGA
CaPYL8 Rev	TAAGTCTGTGATCCCCGCCA
CaSnRK2.3 For	TCTTTGTTGGGGATCCGGC
CaSnRK2.3 Rev	CCATCAAGTCCGCTGGAAG
CaSnRK2.4 For	AGCTGGCTGATGTTTGGTCA
CaSnRK2.4 Rev	AGTTCTTCGGATCCTCCTGG
CaSnRK2.6 For	CCCTGCACCAAGGCTAAAGA
CaSnRK2.6 Rev	TGCAGGTGTACCAACAGTCG

Table S2. Primers used for Y2H plasmid constructs

**Name**	**Sequence (5'-3')**
EcoRI *CaABI* For	AGAATTCATGTTTCCAACACTTGCTGT
XhoI *CaABI* Rev	ATATCTCGAGTTAAATGAAATGCAAGGATGGT
SmaI *CaHAI* For	TCCCCCGGGAATGGCAGAGGTTTGTTTTGGA
XhoI *CaHAI* Rev	ATATCTCGAGCTAGCTCTCCGTTTTGTTCTT
SmaI *CaPYL2* For	TCCCCCGGGAATGGATGGTGATGGCCAATT
PstI *CaPYL2* Rev	AACTGCAGTTATTCGTGCCCATGCAAT
SmaI *CaPYL4* For	TCCCCCGGGAATGCCGATACCGTGTTCAA
PstI *CaPYL4* Rev	AACTGCAGTCACGAAGATTTTTGTGTGA
EcoRI *CaPYL8* For	AGAATTCATGAATGCTAATGGATTTA
BamHI *CaPYL8* Rev	ATGGATCC TTAGACCTGATCAATGGGTT
EcoRI *CaSnRK2.3* For	AGAATTCATGGATCGAGGTCCGGGTCCGT
SalI *CaSnRK2.3* Rev	GCGTCGACTCACATTGCATATATGATCT
EcoRI *CaSnRK2.6* For	AGAATTCATGGATCGGACAGCAGTGACA
BamhI *CaSnRK2.6* Rev	ATGGATCCGAGCACATATTTAGAAGGGAA
